# HLA-DRB5 promotes immune thrombocytopenia via activating CD8^+^ T cells

**DOI:** 10.1515/med-2024-0955

**Published:** 2024-05-23

**Authors:** Qidong Ye, Qianqian Ying, Ying Chen, Cong Liao, Anrong Li

**Affiliations:** Department of Pediatrics, The First Affiliated Hospital of Ningbo University, Haishu District, Ningbo, Zhejiang, 315010, China

**Keywords:** immune thrombocytopenia, cytotoxic T lymphocytes, macrophages, human leukocyte antigen class II heterodimer β5, major histocompatibility complex II

## Abstract

Immune thrombocytopenia (ITP) is an autoimmune disease characterized by a low platelet (PLT) count and a high risk of bleeding, the clinical treatment for which still needs to be upgraded. Based on the critical role of human leukocyte antigen class II heterodimer β5 (HLA-DRB5) in immune system, we herein investigated its effect on ITP. ITP murine models were established by the injection of guinea pig anti-mouse platelet serum (GP-APS), and the PLT of mouse peripheral blood was counted during the modeling. Quantitative real-time reverse transcription polymerase chain reaction, western blot and immunofluorescence assay was performed to quantify expressions of HLA-DRB5, major histocompatibility complex II (MHC-II) and co-stimulatory molecules (CD80, CD86). Flow cytometry was conducted to analyze the percentage of CD8^+^ T cells. As a result, the PLT count was decreased in mouse peripheral blood. Expressions of HLA-DRB5, MHC-II and co-stimulatory molecules, as well as the percentage of CD8^+^ T cells were elevated in peripheral blood of ITP mice. HLA-DRB5 knockdown mitigated ITP by increasing peripheral PLT level, downregulating expressions of MHC-II and co-stimulatory molecules and inactivating CD8^+^ T cells. Collectively, the downregulation of HLA-DRB5 restores the peripheral PLT count in ITP mice by reducing MHC-II-mediated antigen presentation of macrophages to inhibit the activation of CD8^+^ T cells.

## 1 Introduction

Immune thrombocytopenia (ITP), a common acquired autoimmune disorder of the hematologic system, is mainly caused by excessive destruction and decreased production of platelet (PLT) in response to lymphocyte immunity [1]. In addition to common features including a persistently low PLT count (<100 × 10^9^/L) and variable bleeding symptoms, severe ITP patients can develop life-threatening intracranial hemorrhage [2,3]. Currently, corticosteroids, thrombopoietin and immunosuppressants are common clinical therapeutic options, but their efficacy has been limited due to poor response of some patients [4]. Therefore, a comprehensive understanding of the pathogenesis of ITP is of great importance for finding new therapeutic targets and agents to improve patients’ prognosis.

Monocytes/macrophages are a pivotal component of the body’s innate immunity, and can polarize into M1 macrophages and M2 macrophages under stimulation of different factors, affecting the differentiation of lymphocyte subpopulations and altering their immune functions, which are involved in the occurrence and development of many autoimmune diseases [5,6]. It has been confirmed that macrophages play a dual role in the onset of ITP by contributing to PLT destruction and stimulating the adaptive immune response [7,8]. In addition, targeting the imbalance of macrophage polarization is increasingly considered as a potential strategy to suppress ITP [7,9].

Human leukocyte antigen class II heterodimer β5 (HLA-DRB5) is one of the protein-coding loci in tight linkage disequilibrium with HLA-DRB1 and plays an important role in the immune system by presenting peptides to T cells [10]. The dysregulation of HLA-DRB5 has been documented to be associated with the pathogenesis of some autoimmune diseases like systemic lupus erythematosus [11–13]. Previously, the increased expression of HLA-DRB5 has been confirmed in the peripheral leukocytes from children with ITP [14]. But, whether HLA-DRB5 can influence the generation or destruction of PLT remains unknown. During antigen presentation, major histocompatibility complex II (MHC-II) molecules, which are expressed on the surface of antigen-presenting cells such as macrophages and dendritic cells, need to bind to peptide fragments of the antigen for recognition by T cells like CD4^+^ or CD8^+^ T cells [15]. In recent years, the implication of circulating CD8^+^ T cells (cytotoxic T lymphocytes) in ITP pathogenesis has attracted increasing attention given its role in manipulating the fate of PLT [16,17].

In this study, we aimed to identify the effect of HLA-DRB5 on PLT in ITP through detecting surface antigens and PLT count, and to preliminarily explore its potential mechanism. Based on ITP murine models, our current results revealed that the downregulation of HLA-DRB5 inhibits the activation of CD8^+^ T cells to elevate PLT count in peripheral blood of ITP mice, which could be accomplished by decreasing MHC-II-mediated antigen presentation of macrophages.

## 2 Methods

### 2.1 Animals

Six- to eight-week-old BALB/C mice (18–22 g) were housed in an air-controlled room (21–25°C, 50–55% humidity, 12 h on/off light cycles) with available food and drinking water.

### 2.2 Construction of adenovirus

To knock down HLA-DRB5 in mice, ADV3 adenovirus vector was utilized to synthesize short hairpin RNA targeting HLA-DRB5 (sh-HLA-DRB5; sense: 5′-GGTTAGAGTTTATTACAAA-3′, antisense: 5′-TTTGTAATAAACTCTAACC-3′), which was packaged using 293T cells provided by GenePharma (Shanghai, China).

### 2.3 ITP murine modeling and administration

After 1-week acclimation, mice were randomly assigned into four groups (*n* = 6): Control group, ITP group, ITP + vector group and ITP + sh-HLA-DRB5 group. Before establishment of ITP murine models, guinea pig anti-mouse platelet serum (GP-APS) that can be used to induce ITP was customized and purchased from Cloud-Clone Corp. (Wuhan, China). Initially, mice received injection of serum containing GP-APS, and then PLT was removed from the circulation by the fixed phagocyte system [18]. In this study, GP-APS was diluted with saline (G4702, Servicebio, Wuhan, China) in a ratio of 1:4 to prevent erythrocyte adsorption [19]. Then, besides mice from Control group that were given the same volume of saline, mice from other groups were intraperitoneally injected with 100 μL GP-APS every other day. Meanwhile, mice from ITP + sh-HLA-DRB5 group or ITP + vector group were injected with 50 μL diluted sh-HLA-DRB5 adenovirus or negative control by tail vein once a week for 2 weeks. After 15 days, all mice were euthanized by cervical disarticulation under anesthesia (50 mg/kg pentobarbital sodium, P-010; Sigma-Aldrich, St Louis, MO, USA).

### 2.4 Detection of peripheral hemogram

Peripheral blood (20 μL) was sampled from tail vein of each mouse on Days 1, 3, 5, 7, 9, 11, 13 and 15 before GP-APS injection, and diluted with 1× phosphate buffered solution (PBS; abs9459, Absin, Shanghai, China) to a final concentration of 1:7. PLT of the diluted blood was measured using an automatic blood cell counter (XN-3000, Sysmex, Tokyo, Japan) with reference to a previous study [20].

### 2.5 RNA isolation and quantitative real-time polymerase chain reaction (qPCR)

Extraction of total RNA from blood samples was performed using RNeasy Mini Kit (74,104, Qiagen, Hilden, Germany). Complementary DNA was reversely transcribed from equal amounts of total RNA using SuperScript II Reverse Trabscriptase (18,064,014, Thermo Fisher, Waltham, MA, USA). Sequences of primers used in RT-PCR are as follows (5′−3′): HLA-DRB5 (forward: TGGCTGACCTCAGGGACATA, reverse: CCACCAGGCTCCTTACCTTTC), MHC-II (forward: CGTGGTCGGCTGATGGATTT, reverse: GCCATTGTGTGGAGAGTGGA), CD80 (forward: ACCCCCAACATAACTGAGTCT, reverse: TTCCAACCAAGAGAAGCGAGG), CD86 (forward: GGTGGCCTTTTTGACACTCTC, reverse: TGAGGTAGAGGTAGGAGGATCTT) and GAPDH (forward: AGGTCGGTGTGAACGGATTTG, reverse: TGTAGACCATGTAGTTGAGGTCA). Reactions were run on 7900HT Fast Real-Time PCR System (Applied Biosystems, Carlsbad, CA, USA) using SYBR Green Master Mix (4385612, Applied Biosystems, USA). Relative mRNA expressions of specific genes were calculated using 2^−ΔΔCt^ method [21], with normalization to GAPDH.

### 2.6 Western blot

Murine blood samples were homogenized in RIPA Buffer (R0010, Solarbio, Beijing, China) according to the manufacturers’ protocols. Protein concentration of the lysates was determined by BCA Protein Assay Kit (PC0020, Solarbio, China). Equal numbers of protein samples (15 μg/lane) were separated using 10% SDS-PAGE and transferred onto polyvinylidene difluoride membranes (88585, Thermo Fisher, USA), followed by treatment with blocking buffer (P0252, Beyotime, Shanghai, China). Then, primary antibodies against HLA-DRB5 (21702-1-AP, 30 kDa, Proteintech, Chicago, IL, China), MHC-II (ab139365, 29 kDa; Abcam, Cambridge, UK) and GAPDH (ab9485, 36 kDam; Abcam, UK) were used to incubate the membranes at 4°C overnight. The next day, hybridization with horseradish peroxidase-conjugated secondary antibodies (A0192, A0208, Beyotime, China) was performed at room temperature (RT) for 1 h. The stripped membranes were subsequently treated with ECL Western Blotting Detection Kit (SW2040, Solarbio, China), and immunoblots were detected by ChemiDoc XRS + imaging system (BIO-RAD, Hercules, CA, USA). Relative protein expression represented as the ratio of the gray-scale value of the target band to that of the GAPDH band was analyzed by Bandscan software (BIO-RAD, USA).

### 2.7 Immunofluorescence assay

Peripheral blood mononuclear cells (PBMCs) were separated from tail venous blood of mice on Day 15 by density gradient centrifugation in mouse PBMC isolation solution (G2098, Servicebio, China). Next, the cells were fixed with 4% paraformaldehyde (abs9179, Absin, China) for 15 min and permeabilized with 0.1% Triton X-100 (abs47048168, Absin, China) for 10 min at RT, followed by PBS washing and 30 min incubation with 5% bovine serum protein (ST2254, Beyotime, China) at 37°C. Primary antibody against MHC-II (PA5-116876, Thermo Fisher, USA), CD80 (ab254579; Abcam, UK) or CD86 (PA5-114995, Thermo Fisher, USA) was used to incubate the cells at 4°C overnight, and the incubation with fluorescence-labeled secondary bodies (A-11008, A-21244, Thermo Fisher, USA) was performed for 30 min at 37°C. Thereafter, cell nuclei were stained with 2-(4-amidinophenyl)-6-indolecarbamidine dihydrochloride (C1005, Beyotime, China) for 10 min away from light. A confocal microscope (FV3000, Olympus, Tokyo, Japan) was employed to observe the stained cells under ×400 magnification.

### 2.8 Flow cytometry

For immunophenotyping analysis, PBMCs were isolated from tail venous blood of mice on Day 15 by density gradient centrifugation as mentioned above. After PBS washing, FITC-conjugated CD8 antibody (MA1-12028, Thermo Fisher, USA) and PE-conjugated CD4 antibody (MA5-17451, Thermo Fisher, USA) were added into the cell suspension (1 × 10^6^ cells/mL) for 1 h incubation at 4°C in the dark. A flow cytometer (FacsCalibur, BD Biosciences, Franklin Lakes, NJ, USA) was utilized to detect the serum level of CD8^+^ T cells.

### 2.9 Statistical analysis

Data from all triplicate experiments were expressed as mean ± standard deviation. Comparisons between two groups or among multiple groups were carried out using independent-samples *t*-test or one-way analysis of variance. Statistical analysis was completed with GraphPad Prism 8.0 (GraphPad Software Inc., San Diego, CA, USA). A *p* value less than 0.05 was defined to be statistically significant.


**Ethical statement:** Animal experimental procedures in this study were approved by Ethics Committee of Zhejiang Baiyue Biotech Co., Ltd for Experimental Animals Welfare (Approval No. ZJBYLA-IACUC-20221101), and all procedures abided by the guidelines of the China Council on Animal Care and Use.

## 3 Results

### 3.1 HLA-DRB5 and MHC-II were highly expressed in peripheral blood of ITP mice

**Figure 1:**
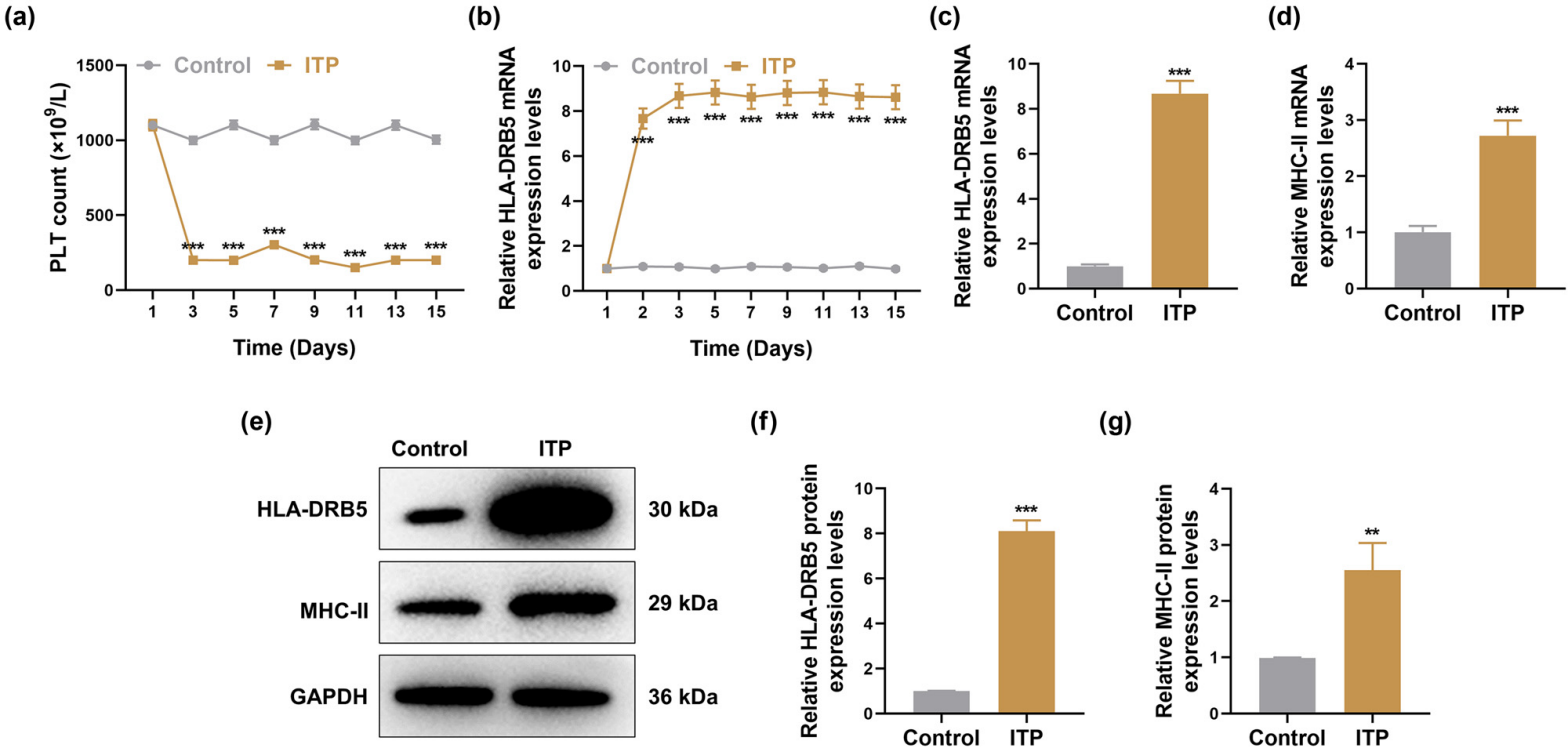
Expressions of HLA-DRB5 and MHC-II in peripheral blood of ITP mice. (a) and (b) ITP murine models were constructed for 15 days. PLT count in peripheral blood was measured every 2 days from the first day, and HLA-DRB5 mRNA expression in peripheral blood was measured by qRT-PCR from the first day (Days 1, 2, 3, 5, 7, 9, 11, 13, 15). (c) and (d) After the ITP modeling for 15 days, analysis of HLA-DRB5 mRNA expression in peripheral blood was performed by qRT-PCR. (e)–(g) Western blot was used to measure HLA-DRB5 protein expression in peripheral blood of ITP murine models. Relative expression was normalized to GAPDH. Data from all triplicate experiments were expressed as mean ± standard deviation. Comparison between two groups was carried out using independent-samples *t*-test. ^**^ *p* < 0.01, ^***^ *p* < 0.001, vs Control. Abbreviation: ITP, immune thrombocytopenia; HLA-DRB5, human leukocyte antigen class II heterodimer β5; qRT-PCR, quantitative real-time reverse transcription polymerase chain reaction.

For mimicking *in vivo* ITP, BALB/C mice received GP-APS injection every other day within 15 days. According to the analysis of automatic blood cell counter, the PLT count of mouse tail venous blood was significantly decreased from Day 3 to Day 5 in the ITP group compared to the Control group (Figure 1a, *p* < 0.001). In addition, HLA-DRB5 mRNA expression in the mouse peripheral blood was markedly increased from Day 2 to Day 3 in the ITP group in comparison to the Control group (Figure 1b, *p* < 0.001). After 15 days, tail venous blood was collected from mice and subjected to qRT-PCR. As demonstrated in Figure 1c and d, both mRNA expressions of HLA-DRB5 and MHC-II were increased in the ITP group relative to the Control group (*p* < 0.001). Consistently, the increased protein levels of HLA-DRB5 and MHC-II were observed in peripheral blood of ITP mice, as determined by western blot (Figure 1e–g, *p* < 0.01).

### 3.2 HLA-DRB5 knockdown increased PLT count and suppressed expressions of MHC-II, CD80 and CD86 in peripheral blood of ITP mice

**Figure 2:**
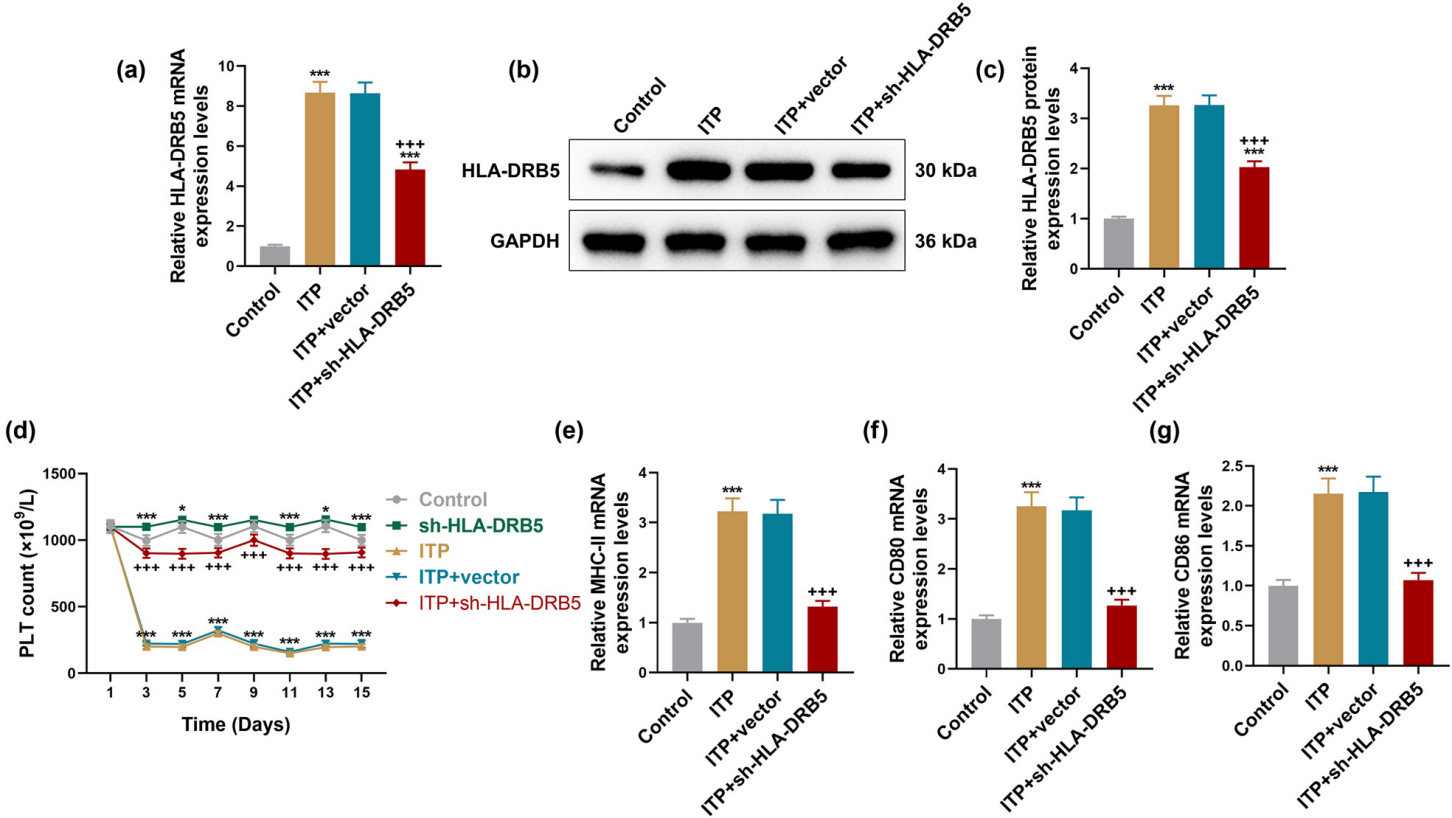
Effects of HLA-DRB5 knockdown on PLT count and expressions of MHC-II, CD80 and CD86 in peripheral blood of ITP mice. (a) ITP murine models were constructed for 15 days, during which sh-HLA-DRB5 adenovirus or negative control was injected into mice by tail vein once a week for 2 weeks. After the ITP modeling for 15 days, analysis of HLA-DRB5 mRNA expression in peripheral blood was performed by qRT-PCR. (b) and (c) Western blot was used to measure HLA-DRB5 protein expression in peripheral blood of ITP murine models. Relative expression was normalized to GAPDH. (d) PLT count in peripheral blood was measured every 2 days from the first day. (e)–(g) QRT-PCR was utilized to detect MHC-II, CD80 and CD86 mRNA expressions in peripheral blood of ITP murine models. Relative expression was normalized to GAPDH. Data from all triplicate experiments were expressed as mean ± standard deviation. Comparison among multiple groups was carried out using one-way analysis of variance. ^*^ *p* < 0.05, ^***^ *p* < 0.001, vs Control; ^+++^ *p* < 0.001, vs ITP + vector. Abbreviation: sh-HLA-DRB5, short hairpin RNA targeting HLA-DRB5.

**Figure 3:**
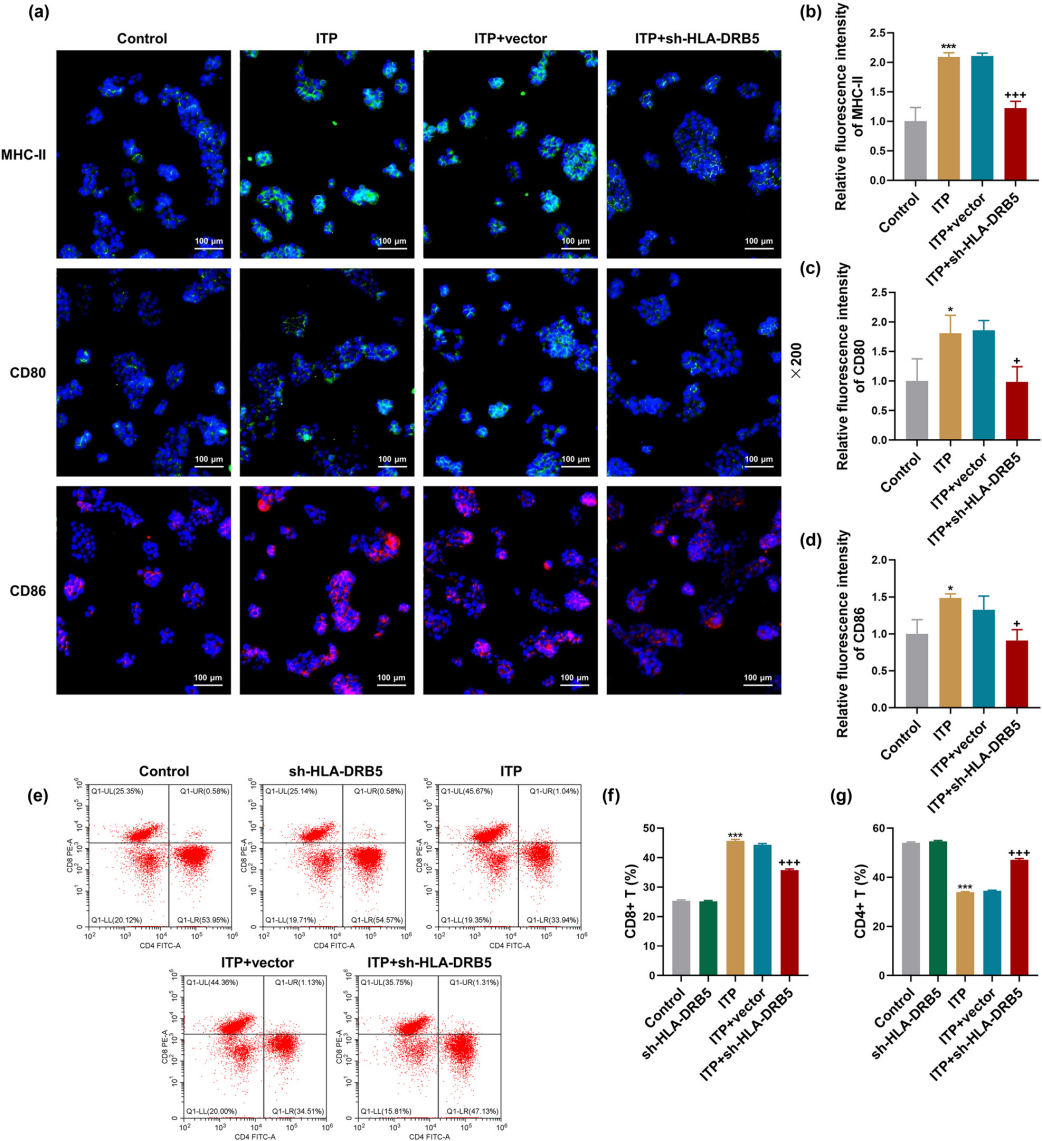
Effects of HLA-DRB5 knockdown on the population of PBMCs with MHC-II^+^, CD80^+^, CD86^+^ and CD8^+^ in ITP mice. (a)–(d) ITP murine models were constructed for 15 days, during which sh-HLA-DRB5 adenovirus or negative control was injected into mice by tail vein once a week for 2 weeks. Fluorescent analysis of MHC-II, CD80 and CD86 in PBMCs of ITP mice was performed using fluorescence-labeled antibodies in combination with fluorescent microscopy (magnification: ×200, scale bar: 100 μm). (e)–(g) Flow cytometry combined with fluorescence-labeled CD8 and CD4 antibodies was performed to detect the percentage of CD8^+^ T cells and CD4^+^ T cells in PBMCs of ITP mice. Data from all triplicate experiments were expressed as mean ± standard deviation. Comparison among multiple groups was carried out using one-way analysis of variance. ^*^ *p* < 0.05, ^***^ *p* < 0.001, vs Control; ^+^ *p* < 0.05, ^+++^ *p* < 0.001, vs ITP + vector. Abbreviation: MHC-II, major histocompatibility complex II; PBMCs, peripheral blood mononuclear cells.

To explore the effect of HLA-DRB5 on ITP, we knocked down HLA-DRB5 in ITP mice through tail vein injection with sh-HLA-DRB5 adenovirus, which resulted in the decreased HLA-DRB5 mRNA and protein expressions in ITP mice (Figure 2a–c, *p* < 0.001). It was observed that sh-HLA-DRB5 increased the PLT count in the Control group mice (Figure 2d, *p* < 0.05), and PLT count was more in the ITP + sh-HLA-DRB5 group than in the ITP + vector group (Figure 2d, *p* < 0.001). Of note, the increased mRNA expressions of MHC-II, CD80 and CD86 in peripheral blood of ITP mice were suppressed by knockdown HLA-DRB5 (Figure 2e–g, *p* < 0.001). We detected the populations of PBMCs with MHC-II^+^, CD80^+^ or CD86^+^ using immunofluorescence assay after mice underwent ITP modeling and/or HLA-DRB5 knockdown. As illustrated in Figure 3a, positive expressions of MHC-II, CD80 and CD86 were increased in PBMCs of ITP mice (Figure 3b–d, *p* < 0.05), which were reversed in PBMCs of ITP mice with HLA-DRB5 deficiency (Figure 3b–d, *p* < 0.05).

### 3.3 HLA-DRB5 knockdown decreased CD8^+^ T cells in peripheral blood of ITP mice

Given the importance of CD8^+^ T cells in destructing PLT in ITP patients [16], we subsequently investigated the effect of HLA-DRB5 on CD8^+^ T cell generation in peripheral blood of ITP mice. The results of flow cytometry showed that the percentage of CD8^+^ T cells was elevated but CD4^+^ T cell percentage was lowered in the ITP group in contrast to the Control group (Figure 3e–g, *p* < 0.001) and interestingly, ITP-induced increase of CD8^+^ T cells and decrease of CD4^+^ T cells in mouse peripheral blood was reversed in the absence of HLA-DRB5 (Figure 3e–g, *p* < 0.001).

## 4 Discussion

For a long time, ITP has been distinguished as two forms: childhood ITP (short-term) and adult ITP (long-last) [2]. Although first-line therapy (e.g., corticosteroids) and second-line therapy (e.g., splenectomy) are effective in most patients with ITP, severe ITP has a high risk of recurrence and bleeding, and is a major cause of death [20,22]. The pathophysiology of ITP has not been fully defined, but its close association with immune cell-mediated loss of immune tolerance in the body has been confirmed in recent reviews.

It is evidenced that PLT autoantibodies are present in about 70% ITP patients, which bind to autoantigens (GPs IIb/IIIa, GPIbIX) on the PLT surface and lead to premature clearance of antibody-opsonized PLT by splenic macrophages or dendritic cells [17]. In some severe ITPs that treatment or treatment escalation is required, CD8^+^ T cells are strongly associated with the severity of thrombocytopenia in the absence of detectible antiplatelet autoantibodies, suggesting that this lymphocyte lineage plays a significant pathogenic role in ITP [23,24]. As a cytotoxic T cells, CD8^+^ T cells has been reported to induce PLT apoptosis when co-cultured with PLT by upregulating proteins like perforin or granzyme B [25]. In this study, we injected GP-APS into mice every other day to construct ITP models and detected PLT count of peripheral blood. Consistent with the results of Zhang et al. [18], we found that the peripheral PLT count of mice was markedly decreased at Day 3 and maintained a low level throughout the modeling process. Of note, we also observed a significant increase of CD8^+^ T cells in peripheral blood of ITP mice. At present, the molecular mechanisms that regulate CD8^+^ T cell-mediated peripheral PLT in ITP remains unclear and needs to be further elucidated.

With the advance of RNA sequencing technology, differentially expressed genes (DEGs) are implicated in the pathophysiology of ITP through regulation of cell functions [26]. Previously, we analyzed DEGs in blood leukocytes from ITP patients and found a significant upregulation of HLA-DRB5, indicating that HLA-DRB5 may play a role in the progression of ITP [14]. In the immune system, HLA-DRB5 is involved in antigen presentation by coding MHC-II to affect immune cell function [27]. Intriguingly, we determined both increased expressions of HLA-DRB5 and MHC-II in peripheral blood of ITP murine models, hinting that HLA-DRB5 could contribute to peripheral PLT destruction in ITP by activating CD8^+^ T cells. In addition to participating in PLT clearance by exerting phagocytosis, macrophages are considered as important antigen-presenting cells to stimulate autoreactive T cells in ITP [28]. It has been demonstrated that ITP macrophages exhibit more M1-related characteristics [29]. In an inflammatory environment stimulated by pathogen, co-stimulatory molecules (CD80, CD86) that are expressed on the surface of M1 macrophages have been suggested to increase cytotoxic T cells by binding to their co-receptors and thereby augment MHC-mediated peptide presentation [30]. Although overexpression of CD80 and CD86 can also be detected in dendritic cells, antigen-presentation-mediated activation and proliferation of T cells in ITP is more dependent on macrophages while dendritic cells should first be loaded with GPIIb/IIIa-trypsin-digested peptides [1]. In this study, the results of qRT-PCR and immunofluorescence assay confirmed that MHC-II, CD80 and CD86 expressions were increased in PBMCs of ITP mice, and their positive expressions were observed on the cell surface. Therefore, it is reasonable to believe that the toxicity of CD8^+^ T cells to PLT of peripheral blood in ITP is activated by macrophage-mediated antigen presentation. To further verify whether HLA-DRB5 can regulate peripheral PLT via CD8^+^ T cell activation, we performed HLA-DRB5 knockdown in ITP murine models. According to the results of rescue assays, it was expectedly observed that HLA-DRB5 knockdown increased PLT count, suppressed expressions of MHC-II, CD80 and CD86, and reduced the percentage of CD8^+^ T cells in peripheral blood. However, the off-target effect of sh-HLA-DRB5 has not been excluded in our study, and we will conduct more rescue experiments to exclude its off-target effect in the future.

## 5 Conclusion

In conclusion, this study provides evidence that HLA-DRB5 may serve as a contributor to the CD8^+^ T cell-induced destruction of peripheral PLT in ITP. Mechanically, our current findings demonstrate that the downregulation of HLA-DRB5 can restore PLT count to normal levels in ITP mice by impairing antigen presentation of macrophage to suppress the activation of CD8^+^ T cells. Therefore, it is indicated that HLA-DRB5 is a potential diagnostic biomarker for ITP, and targeting HLA-DRB5 might be a promising therapeutic strategy to delay the progression of ITP.
